# The basic helix-loop-helix transcription factor, Mist1, induces maturation of mouse fetal hepatoblasts

**DOI:** 10.1038/srep14989

**Published:** 2015-10-12

**Authors:** Hiromi Chikada, Keiichi Ito, Ayaka Yanagida, Hiromitsu Nakauchi, Akihide Kamiya

**Affiliations:** 1 Department of Molecular Life Sciences, Tokai University School of Medicine, 143 Shimokasuya, Isehara, Kanagawa 259-1193, Japan; 2 Division of Stem Cell Therapy, Center for Stem Cell and Regenerative Medicine, The Institute of Medical Science, The University of Tokyo, 4-6-4 Shirokanedai, Minato-ku, Tokyo 108-8639, Japan; 3 Institute for Stem Cell Biology and Regenerative Medicine, Stanford University School of Medicine, 265 Campus Drive, Stanford, California 94305-5461, USA

## Abstract

Hepatic stem/progenitor cells, hepatoblasts, have a high proliferative ability and can differentiate into mature hepatocytes and cholangiocytes. Therefore, these cells are considered to be useful for regenerative medicine and drug screening for liver diseases. However, it is problem that *in vitro* maturation of hepatoblasts is insufficient in the present culture system. In this study, a novel regulator to induce hepatic differentiation was identified and the molecular function of this factor was examined in embryonic day 13 hepatoblast culture with maturation factor, oncostatin M and extracellular matrices. Overexpression of the basic helix-loop-helix type transcription factor, Mist1, induced expression of mature hepatocytic markers such as carbamoyl-phosphate synthetase1 and several cytochrome P450 (CYP) genes in this culture system. In contrast, Mist1 suppressed expression of cholangiocytic markers such as Sox9, Sox17, Ck19, and Grhl2. CYP3A metabolic activity was significantly induced by Mist1 in this hepatoblast culture. In addition, Mist1 induced liver-enriched transcription factors, CCAAT/enhancer-binding protein α and Hepatocyte nuclear factor 1α, which are known to be involved in liver functions. These results suggest that Mist1 partially induces mature hepatocytic expression and function accompanied by the down-regulation of cholangiocytic markers.

The liver is the central organ for metabolism and it performs various functions, such as exocrine drug degradation, nutrition storage, and plasma protein synthesis. Hepatocytes are parenchymal cells that express metabolic enzymes and play important roles in several liver functions^1^,^2^. Other types of cells in the liver, such as cholangiocytes, stellate cells, sinusoidal endothelial cells, mesenchymal cells, and Kupffer cells, are non-parenchymal cells. These cells regulate liver function through cell–cell interaction and soluble factor secretion. In particular, cholangiocytes, which are components of the bile duct, are important for the transport of bile acid^3^. Mature hepatocytes secret bile acid into the intestine and gall bladder through the intrahepatic bile ducts, which are bile canaliculi at the apical membrane of hepatocytes, and interlobular bile ducts derived from cholangiocytes. Hepatocytes and cholangiocytes are both differentiated from hepatic stem/progenitor cells. Liver transplantation is considered to be an effective treatment for end-stage liver diseases. However, it is limited by the shortage of suitable donor organs, the risk of rejections, infections, and lifelong immunosuppression. Hepatocyte transplantation therapy is considered as one of the effective therapies that can substitute for liver transplantation^4^,^5^. In addition, hepatocytes are useful for drug metabolism and pharmacokinetic analyses^6^. A large number of hepatocytes are needed for these purposed; however, hepatocytes are not able to proliferate *in vitro* despite their high proliferation *in vivo*^7^. Hepatoblasts, fetal hepatic stem/progenitor cells, greatly proliferate *in vitro* and differentiate into both mature hepatocytes and cholangiocytes^8^. It would be useful to establish an efficient system to induce differentiation of hepatoblasts into mature hepatocyte-like cells followed by expansion *in vitro*. Previous studies described the induction of hepatic stem/progenitor cells from human iPS cells or ES cells^9^,^10^,^11^, and the isolation of stem/progenitor cells from adult livers^12^,^13^.

During liver development, several functional genes such as tyrosine aminotransferase (TAT)^1^ and several cytochrome P450s (CYPs)^2^ are expressed to obtain mature liver function. CYPs play important roles in the detoxification of drugs and other xenobiotics^14^. Expression of these functional genes is regulated by extracellular signals such as hormones, cytokines, and extracellular matrices. For examples, oncostatin M produced from fetal liver hematopoietic cells has been shown to induce expression of functional genes in fetal hepatic cells^15^,^16^. In addition, previous studies have suggested that hepatocyte nuclear factors (HNFs) 1α, 1β, 3α, 3β, and γ, 4α, and 6 as well as the CCAAT/enhancer-binding protein (C/EBP) family (α, β, and γ) are involved in acquiring hepatocyte functions *in vivo*^17^,^18^. Some of these transcription factors are also used to differentiate pluripotent stem cells^19^,^20^ and somatic cells^21^,^22^,^23^ into hepatocyte-like cells. However, methods to differentiate these cells toward mature hepatocytes have not been developed because expression and functional levels of progenitor-derived cells were significantly lower than those of adult hepatocytes^15^,^24^. We assume that as yet unknown factors are required to effectively promote hepatoblast differentiation toward mature hepatocyte-like cells. In this regard, identification of a novel regulator of hepatocyte maturation could provide an effective method to obtain highly functional hepatocyte-like cells derived from human somatic and pluripotent stem cells.

Basic helix-loop-helix (bHLH) transcription factor Mist1 was first identified as one of the bHLH transcription factor that binds to an E-box sequence using the yeast one-hybrid screening^25^. Mist1 was reported to play an important role in pancreatic acinar cell organization in a study using knockout mice^26^. It is suggested that Mist1 gene locus is activated in both pancreatic and liver organs^27^. However, the function of Mist1 in the liver is still not known. In this study, we found that expression of Mist1 varied throughout liver development. Therefore, we analysed the function of Mist1 in hepatic maturation using an *in vitro* hepatoblast culture system^15^,^28^. Overexpression of Mist1 in embryonic day 13 (E13) hepatoblasts increased the expression of functional genes such as carbamoyl-phosphate synthetase1 (Cps1) and several Cyp genes. In addition, the enzymatic activity of CYP 3A was significantly induced by Mist1 overexpression. In contrast, expression levels of the cholangiocytic markers such as Sox9, Sox17, Ck19, and Grhl2 were decreased with overexpression of Mist1. These data indicate that Mist1 is a novel transcriptional factor that is involved in hepatoblast maturation.

## Results

### Expression of endogenous Mist1 during mouse liver development

**Figure 1:**
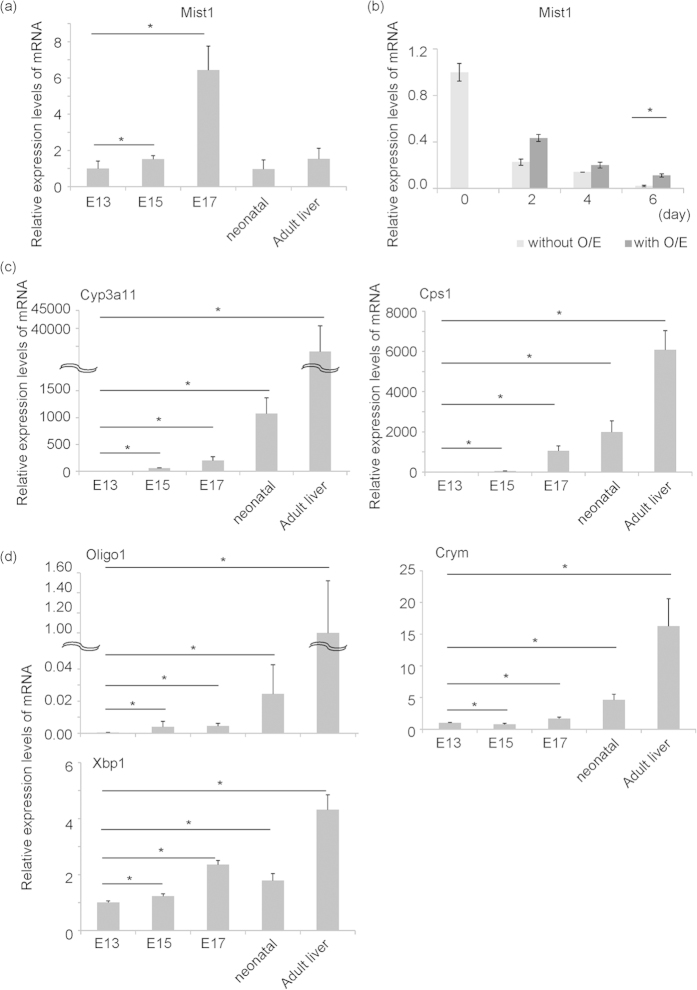
Expression of endogenous Mist1, hepatocytic markers (Cyp3a11 and Cps1) and Mist1-related genes (Oligo1, Crym, and Xbp1) during mouse liver development. (**a,c,d**) Total RNA samples derived from E13, 15, 17, neonatal, and adult livers were purified. Expression of Mist1, Cyp3a11, Cps1, Oligo1, Crym, and Xbp1 was quantified by real-time PCR. Expression of Hprt was used as an internal control. Results are represented as mean expression ± S.D. (n = 8 except for neonatal liver, n = 7 for neonatal liver). *P < 0.05. (**b**) Total RNA samples were purified from E13 fetal hepatoblasts cultured with or without OSM and EHS gel at 0, 2, 4, 6 day (OSM was added at 0 and 2 day, OSM and EHS gel was added at day 4. Expression of Mist1 was quantified by real-time PCR. Expression of Hprt was used as an internal control. Results are represented as the mean expression ± S.D. (n = 3). This experiment was repeated twice independently. O/E: with oncostatin M and EHS gel.

Differentiation and maturation of stem/progenitor cells are regulated by several transcription factors. We analysed expression of transcription factors during liver development and picked up several candidate genes that might regulate maturation of fetal hepatoblasts *in vitro*. To find the new transcriptional factor regulating liver maturation, we expressed these candidate genes in hepatic progenitor cells proliferating on laminin (HPPL), hepatic progenitor cell line^29^, and checked expression of CYP3a11, the liver functional gene (Supplementary Fig. S1). We found that the bHLH type transcription factor Mist1 could induce expression of CYP3a11 in HPPL. Mist1 was expressed more highly in purified E13 hepatoblasts than in adult hepatocytes (Supplementary Fig. S2). Mist1 mRNA was detected in the whole liver from E13 through the neonatal and adult stages and these expression levels peaked at E17 (Fig. 1a). Oncostatin M (OSM) and Engelbreth−Holm−Swarm (EHS) gel are known to induce the maturation of hepatoblasts into functional hepatocyte-like cells *in vitro*^15^,^28^. While the expression of Mist1 decreased in the culture system without the induction of liver maturation, this decrease was partially attenuated by the induction of hepatic maturation using OSM and EHS gel (Fig. 1b). Transient upregulation of endogenous Mist1 might be involved in the differentiation of hepatoblasts into mature hepatocytes *in vivo*. In contrast, Mist1 expression could not maintain and upregulate *in vitro*, suggesting that maturation of hepatoblasts was insufficient in this culture system.

### Expression of hepatocytic and cholangiocytic markers in hepatoblast culture with overexpression of Mist1

**Figure 2:**
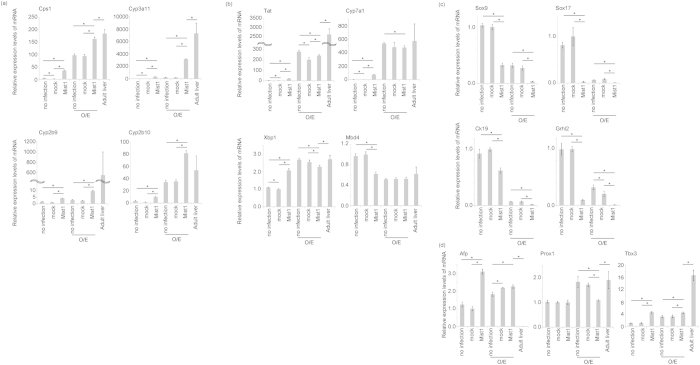
Expression of hepatocytic, cholangiocytic, and fetal hepatocytic markers in hepatoblast culture with overexpression of Mist1. E13 fetal hepatoblasts infected with mock and Mist1-overexpressing viruses were cultured. Simultaneously, E13 fetal hepatoblasts were cultured without infection to confirm that viral infection did not affect liver maturation. When the liver maturation step was finished, cells were collected and total RNA samples were extracted. Moreover, adult livers derived from 8 weeks-old male mice were used as a positive control for liver maturation. (**a**) Expression of hepatocytic markers (Cps1, Cyp3a11, Cyp2b9, and Cyp2b10) was analysed. Expression of these genes was induced by the over-expression of Mist1. (**b**) Expression of hepatocytic markers (Tat and Cyp7a1), Xbp1, and Mbd4 (a negative control) was analysed. Expression of these genes was not induced dramatically by over-expression of Mist1. (**c**) Expression of cholangiocytic markers (Sox9, Sox17, Ck19, and Grhl2) was analysed. (**d**) Expression of fetal markers (Afp, Prox1, and Tbx3) was analysed. (**a–d**) Expression was quantified by real-time PCR. Expression of Tbp was used as an internal control. Results are represented as the mean expression ± S.D. (n = 3 except for adult liver, n = 4 for adult liver). *P < 0.05. This experiment was repeated twice independently.

To reveal the function of Mist1 in liver development, we investigated the effect of Mist1 overexpression on bi-potent differentiation toward hepatocytic and cholangiocytic cells in a hepatoblast culture system. The experimental design of the hepatoblast culture system is shown in Supplementary Fig. S3. We constructed a retrovirus vector expressing mouse Mist1 and overexpression of Mist1 was confirmed by real-time PCR and western blotting (Supplementary Fig. S4). Several metabolism-associated hepatic functional genes are known to be up-regulated during postnatal liver development (Fig. 1c). Overexpression of Mist1 induced the expression of these functional genes, such as Cps1, Cyp3a11, Cyp2b9, and Cyp2b10, in hepatoblast culture with OSM and EHS gel (Fig. 2a). In particular, expression of Cyp3a11 in hepatoblast culture was more than 1000 times lower than that in the adult mouse liver. In contrast, Mist1-induced expression of Cyp3a11 in hepatoblast culture was only 2–3 times lower than that in the adult liver. Some hepatocytic genes, such as Tat and Cyp7a1, could not be induced by the overexpression of Mist1, suggesting that Mist1 partially induced maturation of hepatoblasts (Fig. 2b). Moreover, we found that Mbd4, a negative-control gene in the liver^30^, was not induced by the overexpression of Mist1 in the hepatoblast culture stimulated with OSM and EHS gel (Fig. 2b). In contrast, the expression of cholangiocytic markers such as Sox9, Sox17, Ck19, and Grhl2 were significantly suppressed by overexpression of Mist1 (Fig. 2c).

The bHLH transcription factors have been reported to be necessary for the development of muscles and neurons^31^,^32^. To investigate whether other bHLH transcription factors also have liver maturation activity, we analysed Oligo1, a bHLH transcription factor that is evolutionarily related to Mist1^33^. During liver development, Oligo1 was up-regulated after the postnatal stage (Fig. 1d). In addition, Oligo1 was barely expressed in purified E13 hepatoblasts, but this expression was significantly increased in adult hepatocytes (Supplementary Fig. S5). Thus, we examined Oligo1 activity in the maturation of hepatoblasts using our *in vitro* culture system. As shown above, the overexpression of Mist1 in combination with OSM and EHS gel induced mature hepatocytic markers Cyp3a11 and Cps1. In contrast, overexpression of Oligo1 did not induce expression of these genes.

Hepatoblasts and fetal hepatocytes are known to express several specific marker genes such as α-fetoprotein (Afp), Prox1, and Tbx3. We found that expressions of Prox1 and Tbx3 were highly detected in adult livers; however, AFP was highly expressed in E13-derived hepatoblast culture but not in adult livers. Expression of AFP was not repressed by overexpression of Mist1 in this culture (Fig. 2d), suggesting that other mechanisms were involved in the complete maturation of hepatoblasts.

### Characterization of drug metabolism in mature hepatocyte-like cells derived from E13 hepatoblasts

**Figure 3:**
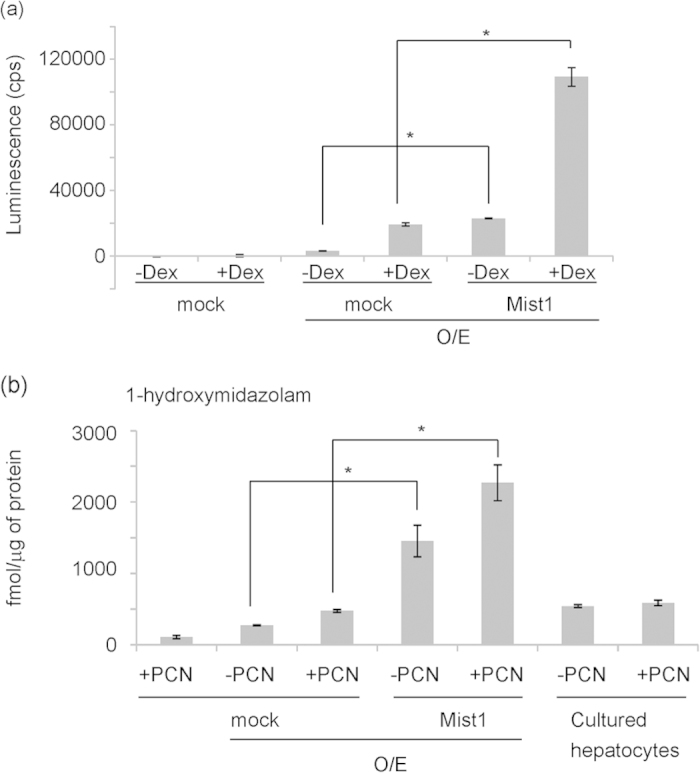
Characterization of drug metabolism in hepatocyte-like cells derived from E13 hepatoblasts. (**a**) CYP3A activity was measured using Luciferin-PFBE. Mock and Mist1-overexpressing hepatoblasts were cultured and hepatic maturation was induced by OSM and EHS gel. The addition of 2 × 10^−4^ M dexamethasone was used for the induction of CYP3A activity. Results are represented as the mean luminescence (cps) ± S.D. (n = 3). *P < 0.05. This experiment was repeated twice independently. (**b**) CYP3A activity was measured using the CYP3A exogenous substrate and this metabolite was quantified by LC-MS/MS. The addition of 1 × 10^−5^ M PCN was used for the induction of CYP3A activity. The cells were incubated with 30 μM midazolam, the CYP3A substrate, for 48 hr. The extracted samples were analysed by LC-MS/MS to quantify the metabolite, 1-hydroxymidazolam. The protein content per well was used to normalize the amount of the metabolite. Results are presented as the mean ± S.D. (n = 3). Cultured hepatocytes: cultured murine adult hepatocytes. *P < 0.05. This experiment was repeated twice independently.

Next, we investigated whether overexpression of Mist1 induced hepatic functions in hepatocyte-like cells derived from E13 hepatoblasts. We focused on drug metabolism through CYP because drug metabolism is one of the most important hepatic functions. The activity of CYP3A, the predominant xenobiotic metabolic enzyme, was analysed in our *in vitro* culture system. Dexamethasone was added to the culture as the CYP3A inducer. Luciferin-PFBE, which was metabolized by CYP3A to produce luciferin, was also added. The amount of luciferin, measured by luciferase assay, was the indicator of CYP3A activity. When hepatic maturation was induced by OSM and EHS gel (**O/E**), CYP3A activity was detected only slightly (Fig. 3a). This activity was significantly up-regulated by the overexpression of Mist1. In addition, CYP3A activity was increased by the addition of dexamethasone.

Next, we directly checked the metabolism of a CYP-target substrate using mass spectrometry. We added the substrate of CYP3A, midazolam, in our *in vitro* culture system after finishing the maturation by OSM and EHS gel (**O/E**). Pregnenolone-16α-carbonitrile (PCN) was used as the inducer of CYP3A. The supernatant was collected and extracted with an organic solvent after a 48-hr incubation with the substrate. This extracted supernatant was analysed to quantify the metabolite of midazolam, 1-hydroxymidazolam, using liquid chromatography-tandem mass spectrometry. At the same time, we prepared cultured adult hepatocytes as a control. When hepatic maturation was induced, 1-hydroxymidazolam was detected at about 270 fmol/μg of protein (Fig. 3b). Overexpression of Mist1 significantly increased the amount of the metabolite to 1450 fmol/μg of protein. Moreover, PCN increased the amount of the metabolite to 470 fmol/μg of protein in control cells and to 2270 fmol/μg of protein in Mist1-overexpressed cells. The amount of the metabolite in adult hepatocytes was less than expected. This was because the cultured hepatocytes lost hepatic function through time. These results suggested that hepatocyte-like cells induced by Mist1 overexpression maintain drug metabolic activity during *in vitro* culture.

### Identification of the molecular mechanism of hepatoblast differentiation by Mist1

**Figure 4:**
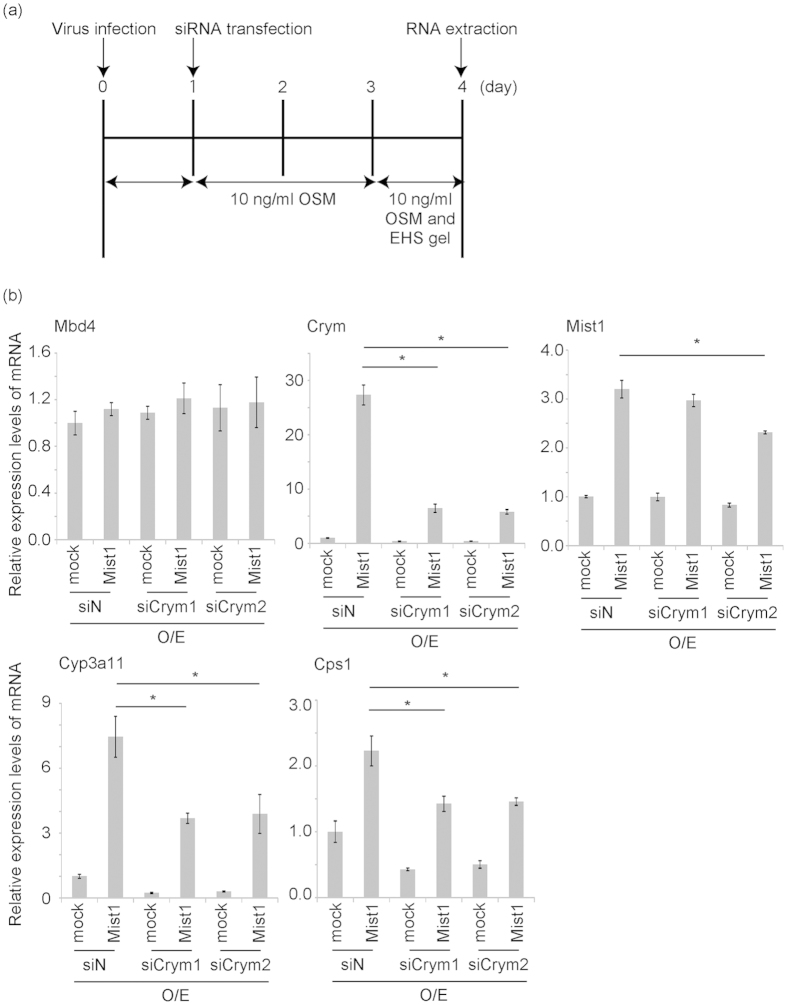
Effect of Crym on hepatic maturation *in vitro* (short term culture). (**a**) The schema of *in vitro* maturation steps with siRNA knock down of short term culture. Fetal hepatoblasts were cultured and retrovirus infection was performed at day 0. Negative control siRNA (siN) and two Crym-specific siRNAs (siCrym1 and siCrym2) are transfected at day 1. Total RNAs were purified at day 4. (**b**) Expression of Mist1 and Crym and hepatocytic markers (Cyp3a11 and Cps1) was quantified by real-time PCR. Expression of Tbp was used as an internal control and expression of Mbd4 was used as a negative control for siRNA transfection. Results are presented as the mean ± S.D. (n = 3). *P < 0.05. This experiment was repeated twice independently.

Next, we determined the expression time course of Cyp3a11 after overexpression of Mist1 in our culture system. The expression of Cyp3a11 was induced after 3–5 days of culture, suggesting that the regulator of hepatoblast maturation is induced by Mist1 in this stage (Supplementary Fig. S6). For the purpose to identify hepatic maturation factors induced by Mist1 in the early culture stage, we evaluated RNA profiles of mock and Mist1-overexpressing hepatoblasts after 3 days of culture using microarray. Among the list of genes that were up-regulated more than 5-fold compared to control hepatoblasts, 253 probes were up-regulated and 70 probes were down-regulated by overexpression of Mist1. To investigate transcriptional regulation by Mist1, we concentrated on nucleus factors induced by Mist1. Of these probes, factors annotated with GO terms containing “transcription” or “nucleus” are shown (Supplementary Fig. S7 and 8). We examined the function of several genes up-regulated by Mist1 by using RNAi knockdown and found that Cyrm, a thyroid hormone binding protein, was partially involved in Mist1-induced hepatic maturation. Knockdown of Crym significantly suppressed the induction of functional genes by Mist1 in short-term culture (4 days, Fig. 4) but not in long-term culture (7 days, Supplementary Figs S9 and S10). These results suggested that Crym is involved only in the early stage of hepatic maturation induced by Mist1.

Interestingly, after peak expression of Mist1 in E17 livers, expression of Crym was up-regulated during postnatal- to adult liver-stages *in vivo* (Fig. 1d). The postnatal to adult livers also expressed several mature hepatocytic markers *in vivo* (Fig. 1c). These results suggest that sequential expression of Mist1 and Crym was correlated with the up-regulation of liver function during postnatal development. It has been reported that expression of Mist1 in zymogenic cells and other cell types is induced by the transcription factor XBP1^34^,^35^, which is involved in liver development^36^; this suggests that XBP1 might also be involved in regulation of Mist1 expression during liver development. Expression of XBP1 was up-regulated in the adult liver (Fig. 1d), but it was not induced by overexpression of Mist1 in the hepatoblast culture stimulated with hepatic maturation factors OSM and EHS gel (Fig. 2b). It has been suggested that Mist1 induced expression of hepatocyte marker genes through the Xbp1 independent manner.

**Figure 5:**
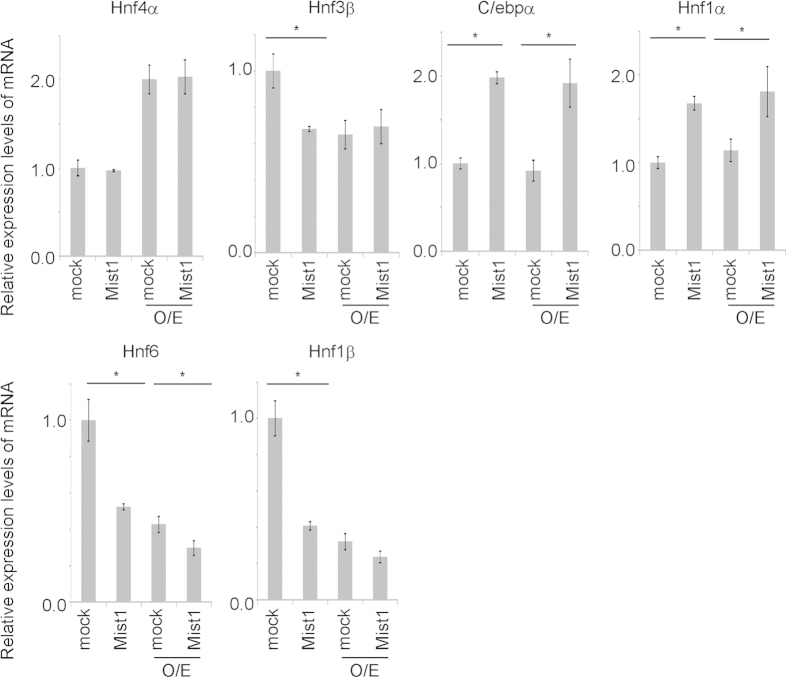
Identification of the molecular mechanism during hepatoblast differentiation by Mist1. The expression of liver-enriched transcription factors was quantified by real-time PCR. Expression of Hprt was used as an internal control. Results are presented as the mean ± S.D. (n = 3). *P < 0.05. This experiment was repeated twice independently.

**Figure 6:**
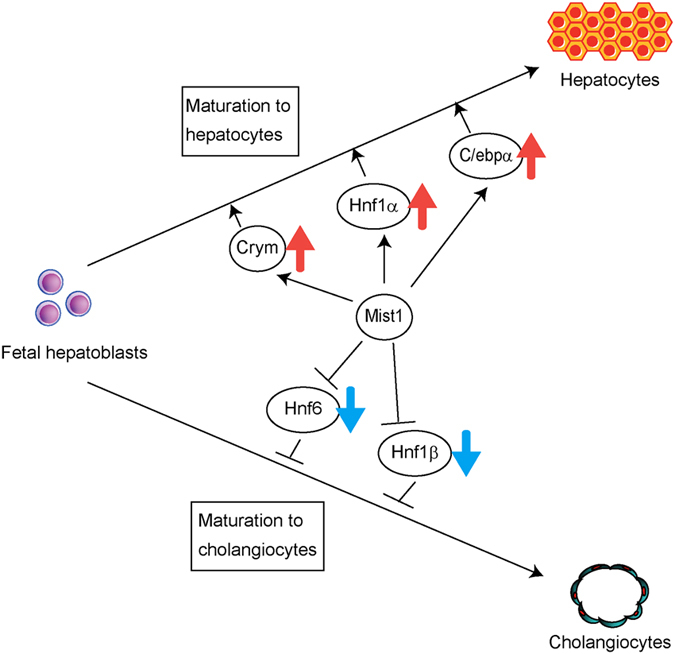
Schematic illustration of this study. The results of this study imply that Mist1 induces liver maturation, which is partly mediated by Mist1-induced Crym expression. However, cholangiocytic maturation was repressed by Mist1.

Expression of functional genes in the liver is regulated by liver-enriched transcription factors, HNFs, and C/EBPs. In contrast, microarray analyses revealed that expression of these factors was not changed significantly by overexpression of Mist1 in the early stage of culture. However, after 6–7 days of culture, the expression of C/ebpα and Hnf1α were significantly induced by the overexpression of Mist1. Conversely, the expression of Hnf6 and Hnf1β was repressed by the overexpression of Mist1 (Fig. 5), suggesting that the differentiation of hepatoblasts into mature hepatocytic cells induced by Mist1 is regulated by changes in these transcriptional factors during long-term culture (Fig. 6).

## Discussion

It has previously been reported that endogenous Mist1 is essential for full maturation of pancreatic acinar cells in Mist1-deficient mice^26^. The function of Mist1 during liver development has not been well-studied, because expression of Mist1 in mid-fetal and adult livers is controversial^26^,^27^,^37^. In this study, we examined the detailed changes in expression of Mist1 in mid- to late-fetal embryonic, perinatal, and adult livers. Expression of Mist1 transiently increased at the perinatal stage (E17 livers), suggesting that the temporal expression of Mist1 is involved in the maturation and fate decision steps of hepatoblasts differentiation into hepatocytes and cholangiocytes. Expression of endogenous Mist1 decreased in a time-dependent manner in the hepatoblast culture. Maturation induced by OSM and EHS gel partially inhibited this reduction. However, the molecular mechanism regulating Mist1 expression remains unknown. The expression of Mist1 is induced at E10.5 in the primitive foregut, caudal, and medial to the forelimb bud^26^. Along pancreatic development, Mist1 expression becomes specific in acinar cells and not in duct or endocrine cell types.

Mist1 is a bHLH transcription factor that binds E-box DNA sequences to regulate the expression of target genes. E-box DNA sequences that Mist1 binds are often located within the first intronic regions^38^,^39^,^40^. Mist1 target genes such as Connexin32, Atp2c2, Rab 26 and 3d and Mindbomb 1 have been identified in the acinar cells or zymogenic cells^38^,^40^,^41^,^42^. We revealed that expression of Cyp3a11 was highly induced by the overexpression of Mist1 in hepatoblast culture. In addition, Cyp2b9 and 2b10 were induced by Mist1. However, we could not find conserved E-box sequences in the promoter or first intronic region of Cyp3a11, Cyp2b9, and Cyp2b10. It may be that Mist1 regulates mature hepatocyte genes indirectly. As shown above, one of the Mist1-inducible genes, Crym, was involved in functional gene expression at an early stage of culture. Crym is reported to be a thyroid hormone binding protein that controls the homeostasis of the thyroid hormone^43^,^44^. A change in thyroid hormone concentration caused by several thyroid diseases sometimes induces liver injury, indicating that Crym may also be involved in liver homeostasis *in vivo*. Suppression of Crym expression did not change hepatic maturation at a late stage in our culture system. At this later stage, several liver-enriched transcription factors were induced by the overexpression of Mist1. These results suggested that Crym partially regulates functional gene expression and that the other target genes regulated by Mist1 are also important for hepatic maturation.

Hepatoblasts have bi-potent differentiation ability; they can differentiate into both mature hepatocytes and cholangiocytes. Time-specific expression of transcription factors determines whether hepatic progenitor cells differentiate to hepatocytes or cholangiocytes^8^. Knockout mice of C/ebpα and Hnf1α showed that these transcription factors are important for hepatocytic differentiation and function^45^,^46^. For examples, deletion of C/ebpα induced differentiation of hepatoblasts into cholangiocytic cells by suppression of hepatocytic differentiation. In this study, we found that C/ebpα and Hnf1α were up-regulated by over-expression of Mist1. In contrast, Hnf6 and Hnf1β, required for normal bile duct formation^47^,^48^, were down-regulated. Thus, it is possible that Mist1 regulates differentiation of hepatoblasts through these transcription factors. In future studies, we shall analyse molecular mechanisms underlying Mist1-associated regulation of these liver-enriched transcription factors.

Our group and other groups have demonstrated methods to differentiate hepatic progenitor cells or pluripotent stem cells into mature hepatocytes^15^,^24^. However, expression levels of mature hepatocytic marker genes in these differentiated cells were significantly lower than in adult hepatocytes. In this study, we found that the overexpression of Mist1 significantly induced expression of several mature hepatocytic marker genes. In particular, the expression level of Cyp3a11, an important drug metabolic gene, was highly induced by Mist1. However, other hepatocytic marker genes was not sufficiently stimulated by overexpression of Mist1. Mist1 is known to bind to E-box elements as a heterodimer with other transcriptional regulators. Interestingly, the Mist1 protein has no functional transcription activator domain, suggesting that heterodimer partner proteins are important for the induction of efficient hepatoblast differentiation in this system^25^. Future analyses of Mist1 and Mist1 partner proteins might enable full maturation of hepatoblasts *in vitro* for drug screening and cell transplantation therapy of severe liver diseases.

## Methods

### Materials

C57BL/6N mice were purchased from Nihon SLC (Shizuoka, Japan). Dulbecco’s modified Eagle’s medium (DMEM), dexamethasone, and gelatin were purchased from Sigma (St Louis, MO, USA). Fetal bovine serum (FBS) was purchased from Nichirei Biosciences (Tokyo, Japan). MEM Non-Essential Amino Acids Solution, Penicillin-Streptomycin-Glutamine and Insulin-Transferrin-Selenium X were purchased from Life Technologies (Carlsbad, CA, USA). Murine OSM was purchased from R&D systems (Minneapolis, MN, USA). EHS gel was purchased from Becton Dickinson (Growth Factor Reduced Matrigel Matrix, Bedford, MA, USA). Collagenase was purchased from Worthington Biochemical Corporation (Lakewood, NJ, USA). Complete Protease Inhibitor Cocktail was purchased from Roche Diagnostics (Basel, Switzerland).

### Animal Experiments

All animal experiments were performed in accordance with the approved guidelines and these protocols were approved by the Institutional Animal Care and Use Committee in both the Institute of Medical Science of the University of Tokyo (permit number: A09–21) and Tokai University (permit number: 144008).

### Isolation of fetal, neonatal, and adult livers

E13, 15, 17, and neonatal livers were excised under microscope. Adult livers derived from 8-week-old male mice were excised after bleeding. Livers were collected into RNA later® solution (Life Technologies) and held at -80 °C before extraction of total RNA.

### Isolation and culture of Dlk^+^ hepatoblasts from fetal liver

Minced embryonic liver tissues from E13 mice were digested with liver perfusion buffer (0.5 mM EGTA solution) and liver digest medium (0.05% collagenase solution). Cells were incubated at 4 °C for 30 min with biotin anti-CD45 and biotin anti-Ter119/Erythroid cells (Biolegend, San Diego, CA, USA). Cells were washed in phosphate-buffered saline (PBS) with 3% FBS (staining medium) and incubated with Dynabeads® MyOne^TM^ streptavidin C1 (Life Technologies). Cells were washed and resuspended in staining medium and hematopoietic cells (CD45^+^Ter119^+^) were eliminated using Dyna Mag^TM^ 15 (Life Technologies). The supernatant was recovered and anti-Dlk antibody (Preadipocyte factor-1^49^, Medical and Biological Laboratories, Nagoya, Japan) was added to the cell pellets. After incubation and washing, cells were incubated with anti-Rat IgG micro beads (Miltenyi biotech, Bergisch Gladbach, Germany). After another wash, cells were resuspended and loaded onto the pre-washed MACS® Separation LS column (Miltenyi biotech). CD45^−^Ter119^−^Dlk^+^ cells were eluted from the column and used as the hepatoblast fraction.

Purified Dlk^+^ hepatoblasts were cultured in hepatocyte culture media (DMEM supplemented with 10% FBS, 1×MEM Non-Essential Amino Acids Solution, 10^−7^ M dexamethasone, and 1× Penicillin-Streptomycin-Glutamine) on 0.1% gelatin-coated tissue culture dishes. Then, OSM and EHS gel were used for hepatic maturation. For the EHS gel overlay, the culture medium was removed and EHS diluted in ice-cold hepatocyte culture media with OSM at a volume ratio of 1:5 (EHS/Medium) was added to the culture dishes. Cells were harvested at the indicated times, depending on each analysis.

### Generation and infection of retroviruses

The retroviral vector pGCDNsam, with a long terminal repeat derived from MSCV, has intact splice donor and splice acceptor sequences for the generation of subgenomic messenger RNA. The complementary DNA (cDNA) of mouse Mist1 was subcloned into an upstream sequence of an internal ribosomal entry site (IRES) in a pGCDNsam vector. This vector has the sequence of enhanced green fluorescent protein downstream from the IRES. Infected cells thus can be detected using a fluorescent microscope. Retroviruses were generated as previously described^50^. Virus titers were determined by infection of NIH3T3. Retrovirus infection was performed 1 hr after plating fetal hepatoblasts. A multiplicity of infection is 5. The next day after infection, the culture medium was replaced with fresh medium.

### mRNA detection by reverse transcription-polymerase chain reaction (RT-PCR)

Total RNA samples were extracted with RNA iso Plus (TAKARA, Shiga, Japan). First-strand cDNA was synthesized using a High-Capacity cDNA Reverse Transcription Kit (Life Technologies) or ReverTra Ace qPCR RT Master Mix with genome remover (TOYOBO, Osaka, Japan) and used as a template for quantitative RT-PCR. The cDNA samples were normalized by the number of hypoxanthine guanine phosphoribosyl transferase (Hprt) copies. The cDNA samples in Fig. 2 and 4 and Supplementary Fig. S4a, S5b, S9, and S10 were normalized by the number of TATA box-binding protein (Tbp) copies. Quantitative analyses of the expression of target mRNAs were performed using the Universal Probe Library System (Roche Diagnostics). Primers and probes for quantitative RT-PCR are shown in Supplementary Table S1.

### Western blot analysis

Fetal hepatoblasts infected with mock or Mist1-overexpressing retrovirus were washed with PBS and lysed with RIPA buffer (20 mM TrisHCl pH7.4, 150 mM NaCl, 1 mM EDTA, 1% Nonidet P-40, 0.1% sodium deoxycholate, 0.1% sodium dodecyl sulfate [SDS], and 1× Complete Protease Inhibitor Cocktail). The protein lysates were mixed with SDS sample loading buffer containing β-mercaptoethanol, electrophoresed on a 15% SDS-polyacrylamide gel, and electrotransferred onto an Immobilon-P membrane (Millipore, Billerica, MA, USA). The membrane was blocked with PBS with Tween 20 (PBS-T) containing 5% Amersham^TM^ ECL^TM^ Blocking Agent (GE healthcare UK Ltd., Backinghamshire, England) overnight, then incubated with primary antibody, Mist1 antibody (Thermoscientific, Waltham, MA, USA), in blocking buffer for 2 hr. Then, the membrane was washed with PBS-T and incubated with a horseradish peroxidase-conjugated secondary antibody (Millipore). After another wash with PBS-T, immunoreactive protein was developed by the ECL reagent (Millipore) and the chemiluminescence was captured with an ATTO Ez-Capture MG AE-9300 (ATTO Corporation, Tokyo, Japan).

### Measurement of CYP 3A activity

Hepatoblasts were infected with mock or Mist1-overexpressing retrovirus and cultured. After finishing the differentiation steps, hepatocyte culture medium with 2 × 10^−4 ^M dexamethasone was added for 24 hr to induce CYP3A expression. Then, the culture medium and EHS gel were removed and cells were washed with PBS. CYP3A activity was measured using the P450-Glo^TM^ CYP3A4 Assay (Luciferin-PFBE) Cell-Based/Biochemical Assay (Promega, Madison, WI, USA) according to manufacturer’s protocol.

In the measurement of CYP3A activity using the LC-MS/MS analysis, fetal hepatoblasts were cultured as described above. After the differentiation step using OSM and EHS gel, hepatocyte culture medium with 1 × 10^−5^ M PCN was added onto the EHS gel for 20 hr to induce the CYP3A activity. Then, the hepatocyte culture medium and EHS gel were removed by washing with PBS. The substrate of CYP3A, 30 μM midazolam (Cerilliant, Round Rock, TX, USA) was added for 48 h. The supernatant was collected and mixed with 2 volumes of methanol/acetonitrile (1:1) to remove proteins. Hepatocytes purified from adult mice were cultured as a positive control. As an internal control, 5-(4-Hydroxyphenyl)-5-phenylhydatoin (Sigma-Aldrich) was used. After removal of protein, the extract was diluted with water (for LC-MS, Sigma-Aldrich). Diluted samples were analysed by LC-MS/MS to quantify the metabolite, 1-hydroxymidazolam, according to the standard curve. The LC-MS/MS system consisting of a Nexera X2 HPLC system (Shimadzu Corporation, Kyoto, Japan) and LCMS-8050 (Shimadzu Corporation) with a Synergi Hydro-RP (50 mm × 2.0 mm i.d., 4 μm; Phenomenex, Torrance, CA, USA) was used for quantification. The mass spectrometer was set to the multiple-reaction monitoring mode and was used with the electrospray ionization source in positive ion mode. The mobile phase was delivered at a flow rate of 0.2 ml/min using a gradient elution profile consisting of solvent A (0.1% acetic acid/distilled water) and solvent B (0.1% acetic acid/methanol). Supplementary Table S2 and S3 showed the details of the LC gradient conditions and mass spectrometer conditions. The metabolite concentration was determined according to each standard (normalized to the protein content per well sample).

### Culture of adult hepatocytes

Adult mice were subjected to a standard two-step collagenase perfusion for the isolation of primary hepatocytes. The liver was pre-perfused through the portal vein with 0.5 mM EGTA solution and perfused with 0.025% collagenase (Yakult, Tokyo, Japan) solution. Hepatocytes were purified by 50% Percoll^TM^ (GE healthcare) buffer at 50 × *g* for 10 min. Primary hepatocytes were cultured in William’s medium E supplemented with 10% FBS, 1×Insulin-Transferrin-Selenium X, 10^−7^ M dexamethasone, and 1× Penicillin-Streptomycin-Glutamine in collagen type 1-coated dishes.

### Transcription profile analysis using microarray

Dlk^+^ hepatoblasts derived from E13 fetal livers were purified as described above. Cells were infected with mock or Mist1-overexpressing retroviruses and cultured for 3 days. Total RNA was purified from these cells using the RNeasy Micro Kit (Qiagen, Limburg, Netherlands), according to the manufacturer’s instructions. Transcription profiles were analysed using the Agilent Whole Mouse Genome Microarray 8 × 60 K. Hierarchical clustering of normalized signal intensities was performed using Euclidean distances and centroid linkage. Raw intensity values were normalized using the 75th percentile and transformed to log2 scale. The original data are available from the Gene Expression Omnibus (accession number GSE67406).

### siRNA transfection in fetal hepatoblasts cultured with OSM and EHS gel

After fetal hepatoblasts were seeded in 24-well 0.1% gelatin-coated tissue culture dishes, the cells were infected with mock or Mist1-expressing retroviruses. siRNA transfection was performed using X-treme Gene siRNA Transfection Reagent (Roche Diagnostics) according to the manufacturer’s protocol. siRNAs were purchased from Dharmacon (Lafayette, CO, USA). The culture and transfection schedule are shown in Fig. 4 and Supplementary Fig. S9 and S10. The cells were harvested at the indicated time for real-time PCR analysis.

### Statistics

Microsoft Excel 2010 (Microsoft, Redmond, WA, USA) was used to calculate standard deviations (SD) and statistically significant differences between samples using a Student’s two-tailed test.

## Additional Information

**How to cite this article**: Chikada, H. *et al.* The basic helix-loop-helix transcription factor, Mist1, induces maturation of mouse fetal hepatoblasts. *Sci. Rep.* **5**, 14989; doi: 10.1038/srep14989 (2015).

## Supplementary Material

Supplementary Information
