# Supplementary material for: The basic helix-loop-helix transcription factor, Mist1, induces maturation of mouse fetal hepatoblasts
Source: Sci Rep. 2015 Oct 12;5:14989. doi: 10.1038/srep14989 (PMC4601036; doi:10.1038/srep14989)
Supplement: Supplementary Information [file srep14989-s1.pdf]

The basic helix-loop-helix transcription factor, Mist1, induces maturation of mouse fetal hepatoblasts

Hiromi Chikada, Keiichi Ito, Ayaka Yanagida, Hiromitsu Nakauchi, and Akihide Kamiya

[Supplementary figure legends]

Supplementary figure S1 Identification of a liver maturation factor in hepatic progenitor cells proliferating on laminin (HPPL). HPPL was cultured and passaged at day 0. Retroviral infection was performed the next day of passage (at day 1). Cells were washed with PBS and exchanged for fresh medium at day 2. After OSM was added at day 2, medium was changed once every two days for a total of two times. At day 6, OSM and EHS gel were added for an additional 2 days of culture. The effect of the candidates on liver maturation was confirmed by quantification of expression of Cyp3a11 (n=1).

Supplementary figure S2 Expression of Mist1 during liver development. Total RNA samples were extracted from E13 purified hepatoblasts and adult hepatocytes. Expression was quantified by real-time PCR. Expression of Hprt was used as an internal control. Results are presented as the mean ± S.D. (n=3). *P<0.05.

Supplementary figure S3 The schema of *in vitro* maturation steps of hepatoblast derived from E13 fetal livers. Fetal hepatoblasts were isolated and cultured at day 0. Retrovirus infection was performed 1 hr after cells were inoculated on gelatin-coated dishes. Cells were washed with PBS and exchanged for fresh medium at day 1. After OSM was added at day 1, medium was changed once every two days for two times total. At day 5, OSM and EHS gel were added for an additional 1-2 days of culture.

Supplementary figure S4 Overexpression of Mist1 in hepatoblast culture. (a) Expression of Mist1 was quantified by real-time PCR. Expression of Tbp was used as an internal control. Results are presented as the mean ± S.D. (n=3 except for adult liver, n=4for adult liver). *P<0.05. This experiment was repeated twice independently. (b) Expression of MIST1 protein. Fetal hepatoblasts were cultured

without OSM and EHS gel. The cells were lysed with RIPA buffer. Expression of MIST1 was detected by western blotting (n=2). Arrow showed the MIST1 protein.

Supplementary figure S5 The effect of Oligo1 during liver development. (a) Expression of Oligo1 during liver development. Total RNA samples were extracted from E13 purified hepatoblasts and adult hepatocytes. Expression was quantified by real-time PCR. Expression of Hprt was used as an internal control. Results are presented as the mean ±S.D. (n=3). *P<0.05.

(b) Effect of Oligo1 on expression of liver functional genes in E13 hepatoblast culture. E13 foetal hepatoblasts infected with mock or Oligo1 or Mist1-overexpressing retrovirus were cultured and hepatic maturation was induced by the addition of OSM and EHS gel. Expression of Cyp3a11 and Cps1 was quantified by real-time PCR. Expression of Tbp was used as an internal control. Results are presented as the mean ± S.D. (n=3). *P<0.05. This experiment was repeated three times independently.

Supplementary figure S6 Expression of Cyp3a11 in fetal hepatoblast culture was quantified under Mist1-overexpression by real-time PCR. Cyp3a11 mRNA expression levels are shown in the graph and the table. This experiment was repeated twice independently.

Supplementary figure S7 Microarray analysis annotated GO terms containing transcription in E13 hepatoblasts infected with mock and Mist1-overexpressing retroviruses. Colours range from bright red (Up-regulation) to bright green (Down-regulation). The heat map shows the genes whose expression levels were changed more than 5-fold by overexpression of Mist1 (n=2).

Supplementary figure S8 Microarray analysis annotated nucleus as a GO term in E13 hepatoblasts infected with mock and Mist1-overexpressing retroviruses. Colours range from bright red (Up-regulation) to bright green (Down-regulation). The heat map shows the genes whose expression levels were changed more than 5-fold by overexpression of Mist1 (n=2).

Supplementary figure S9 Effect of Crym on hepatic maturation *in vitro* (long term culture). (a) The schema of *in vitro* maturation steps with siRNA knock down in long-term culture. Fetal hepatoblasts were cultured and retrovirus infection was performed at day 0. Negative control siRNA (siN) and two Crym-specific siRNAs (siCrym1 and siCrym2) are transfected at day 1 and 3. Total RNAs ware purified at day 7. (b) Expression of Mist1, Crym, and hepatic markers (Cyp3a11 and Cps1) was quantified by real-time PCR. Expression of Tbp was used as an internal control and expression of Mbd4 was used as a negative control for siRNA transfection. Results are presented as the mean ± S.D. (n=3). *P<0.05. This experiment was repeated twice independently.

Supplementary figure S10 Effect of Crym on hepatic maturation *in vitro* (long-term culture with single siRNA transfection). (a) The schema of *in vitro* maturation steps with siRNA knock down in long-term culture. Fetal hepatoblasts were cultured and retrovirus infection was performed at day 0. Negative control siRNA (siN) and two Crym-specific siRNAs (siCrym1 and siCrym2) are transfected at day 3. Total RNAs ware purified at day 7. (b) Expression of Mist1, Crym, and hepatic markers (Cyp3a11 and Cps1) was quantified by real-time PCR. Expression of Tbp was used as an internal control and expression of Mbd4 was used as a negative control for siRNA transfection. Results are presented as the mean ± S.D. (n=3). *P<0.05.

Supplementary Table S1

PCR primers for detection of mouse gene expression

Mouse genes	Forward primer (5'→3')	Reverse primer (5'→3')	Probe number
Hprt	tcctcctcagaccgctttt	cctggttcatcatcgctaatc	95
Tbp	ggcggtttggctaggttt	gggttatcttcacacaccatga	107
Mbd4	caacgactcctaccggatct	tggtatttatttaacttgtggtcttca	38
Mist1	ggctaaagctacgtgtccttg	ggtgaggcccttccaact	110
Tat	ggaggaggtcgcttcctatt	gccactcgtcagaatgacatc	82
Cps1	gacaccactgcccgagac	cagcagacctgccacctt	95
Cyp3a11	gggactcgtaaacatgaacttttt	ccatgtcgaatttccataaacc	53
Cyp2b9	agcgccaccctccactat	caatctccttttggactttctctg	15
Cyp2b10	aagctcattctccagccaga	ctgtgggcaccaggaaag	106
Cyp7a1	tcaagcaaacaccattcctg	ggctgctttcattgcttca	50
Sox9	cagcaagactctgggcaag	tccacgaagggtctcttctc	66
Sox17	cacaacgcagagctaagcaa	cgcttctctgccaaggtc	97
Ck19	tgacctggagatgcagattg	cctcagggcagtaatttcctc	17
Grhl2	ccacagagcatactgccaga	tctcttcatcccggatttttc	32
Afp	catgctgcaaagctgacaa	ctttgcaatggatgctctctt	63
Prox1	cgacatctcaccttattcagga	ttgcctttttcaagtgattgg	4
Tbx3	ttgcaaagggttttcgagac	gactgcagtgtgagctgctt	51
Hnf4α	ccaagaggtccatggtgttt	ccgagggacgatgtagtcat	68
Hnf3β	gagcagcaacatcaccacag	cgtaggccttgaggtccat	77
C/ebpα	aaacaacgcaacgtggaga	gcggtcattgtcactggtc	67
Hnf1α	cgcctccaccctggttat	actccccatgctgttgatg	98
Hnf6	ggagttccagcgcatgtc	cgacgttggacgtctgtg	64
Hnf1β	atggctcccctcaccatc	ggttgtagcgcactcctga	55
Oligo1	ccgcaagctctccaagatt	ctcctgcagcgagctacc	18
Crym	ggggctcacatcaatgct	gctcgtcatccagttctcg	40
Xbp1	agcaagtggtggatttggaa	ccgtgagttttctcccgtaa	78

Supplementary Table S2 Gradient

Time (min)	B Conc. (%)
0.00-0.10	0
0.10-9.00	100
9.00-10.00	100
10.00-10.10	0
10.10-15.00	0

Supplementary table S3 MRM conditions

	Parent (m/z)	Daughter (m/z)	Q1 Pre Bias (V)	Collision energy (V)	Q3 Pre Bias (V)
1-hydroxymidazolam	341.75	324.00	-30.0	-25.0	-30.0

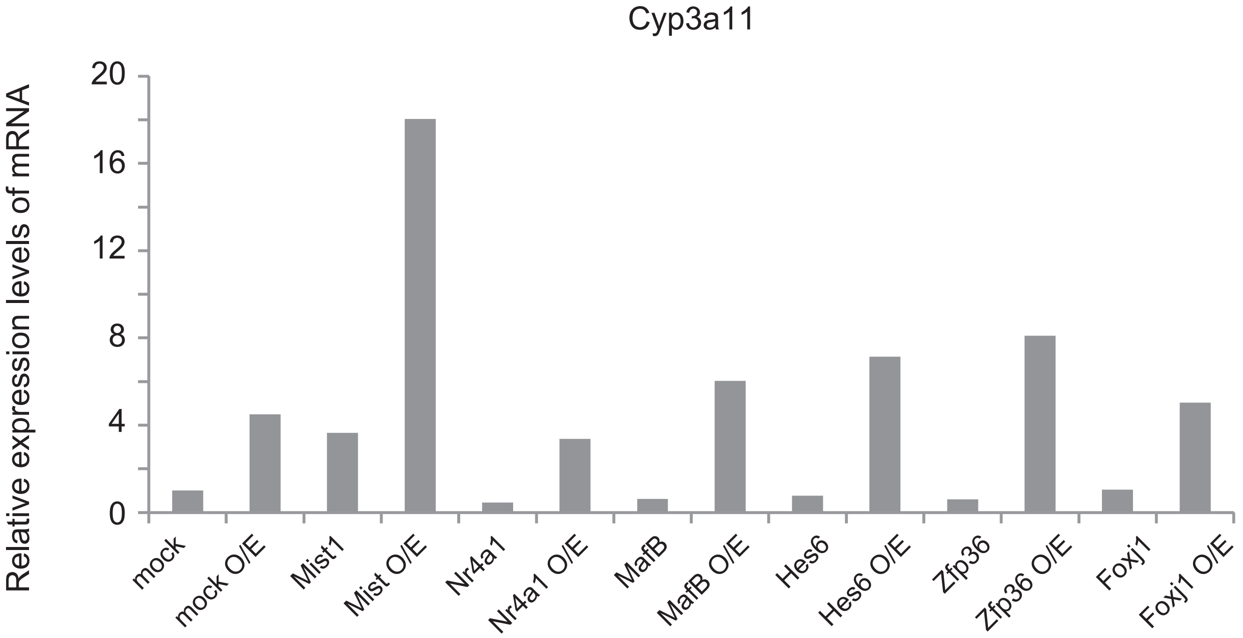

Chikada et al., Supplementary Figure S1

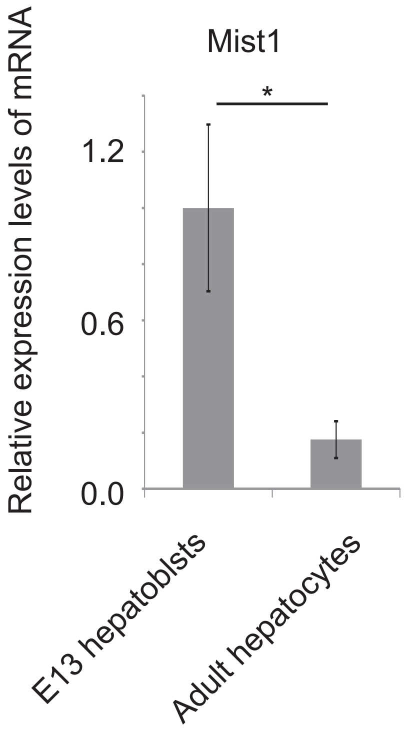

Chikada et al. Supplementary Figure S2

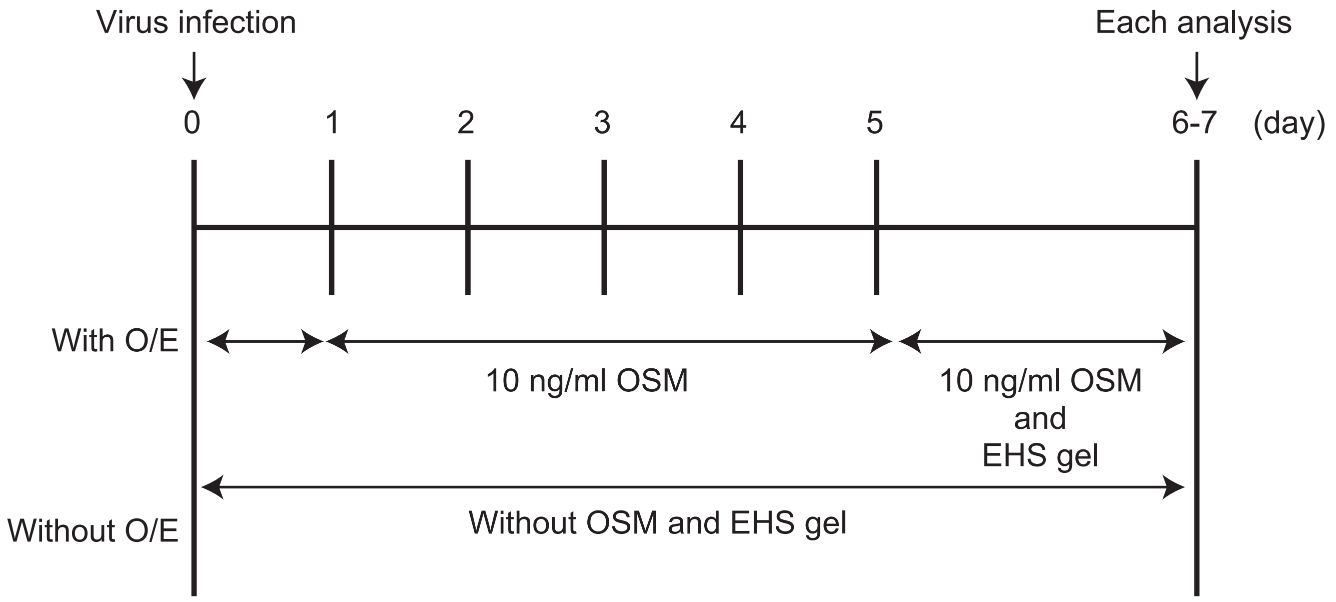

Chikada et al. Supplementary Figure S3

(a)

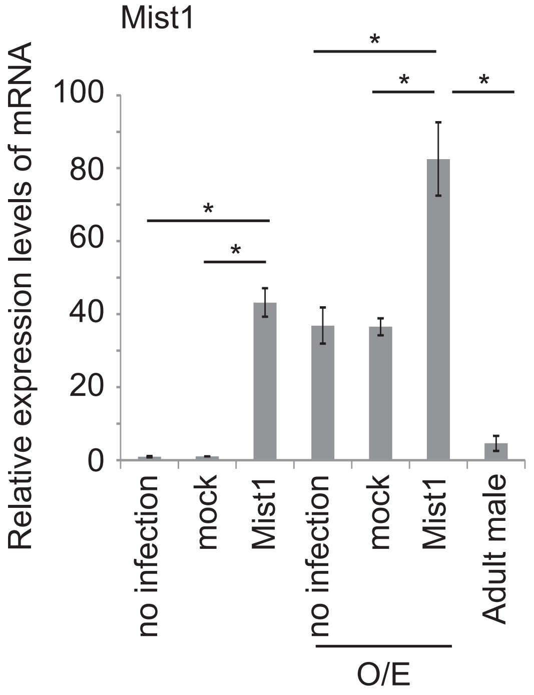

(b)

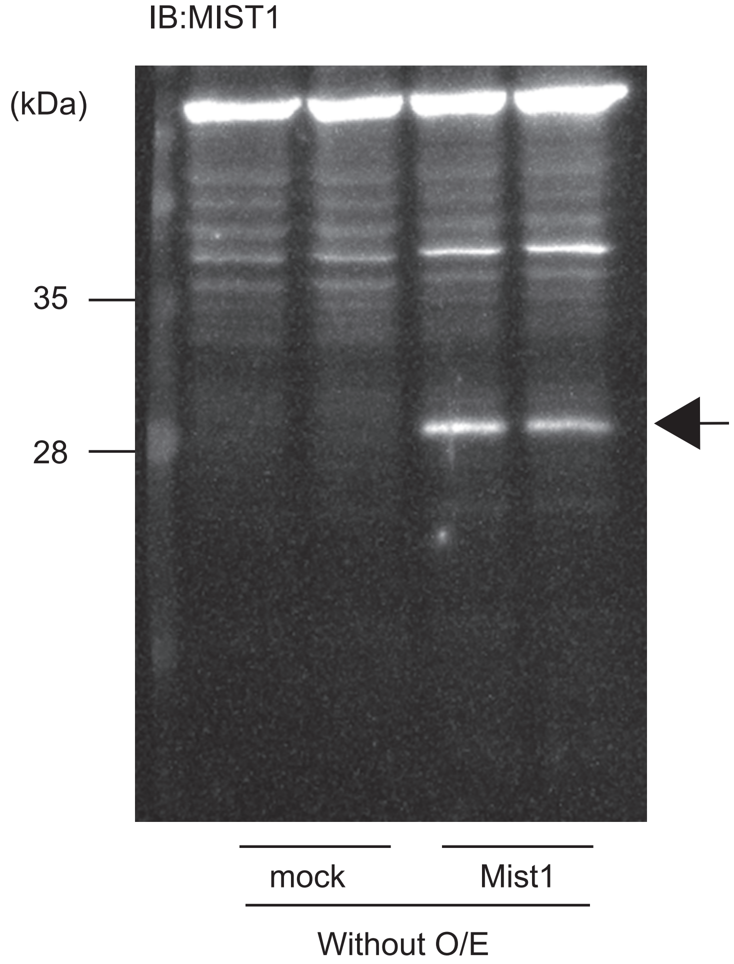

Chikada et al. Supplementary Figure S4

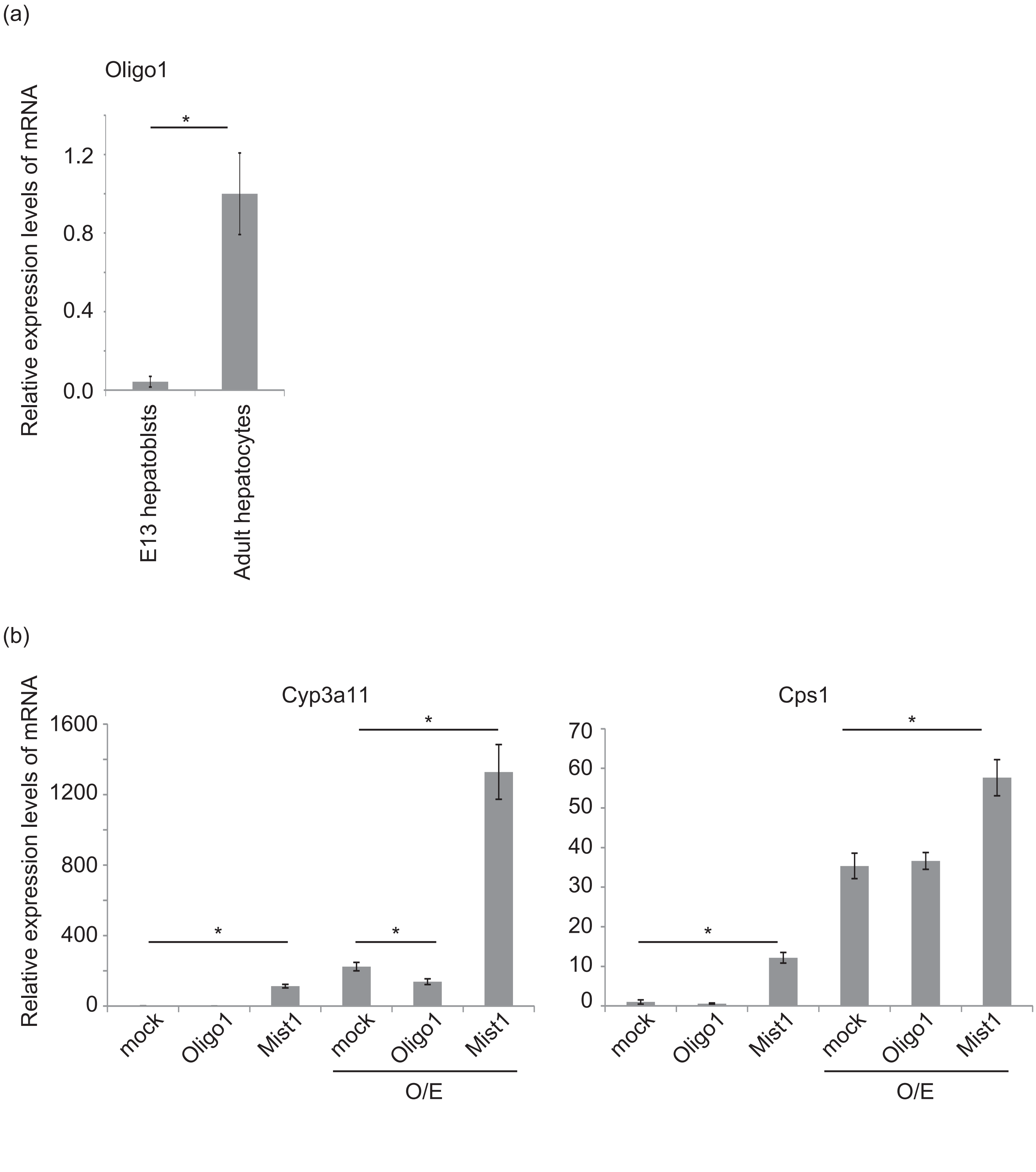

Chikada et al., Supplementary Figure S5

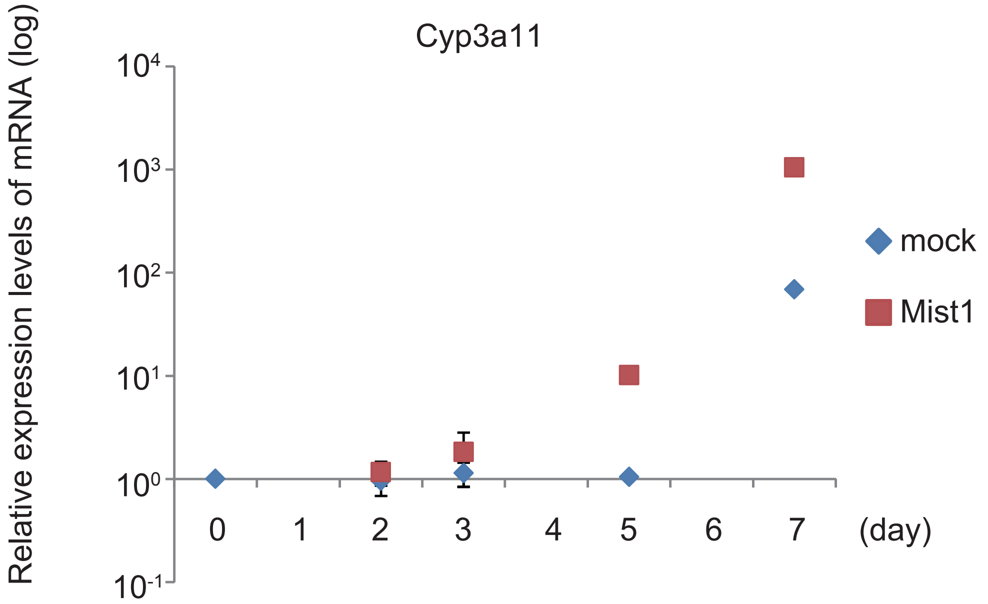

Quantify the Cyp3a11 mRNA expression levels

	0	2	3	5	7 (day)
mock	1.000	0.958	1.140	1.046	68.799
Mist1		1.163	1.823	10.165	1043.842

Chikada et al. Supplementary Figure S6

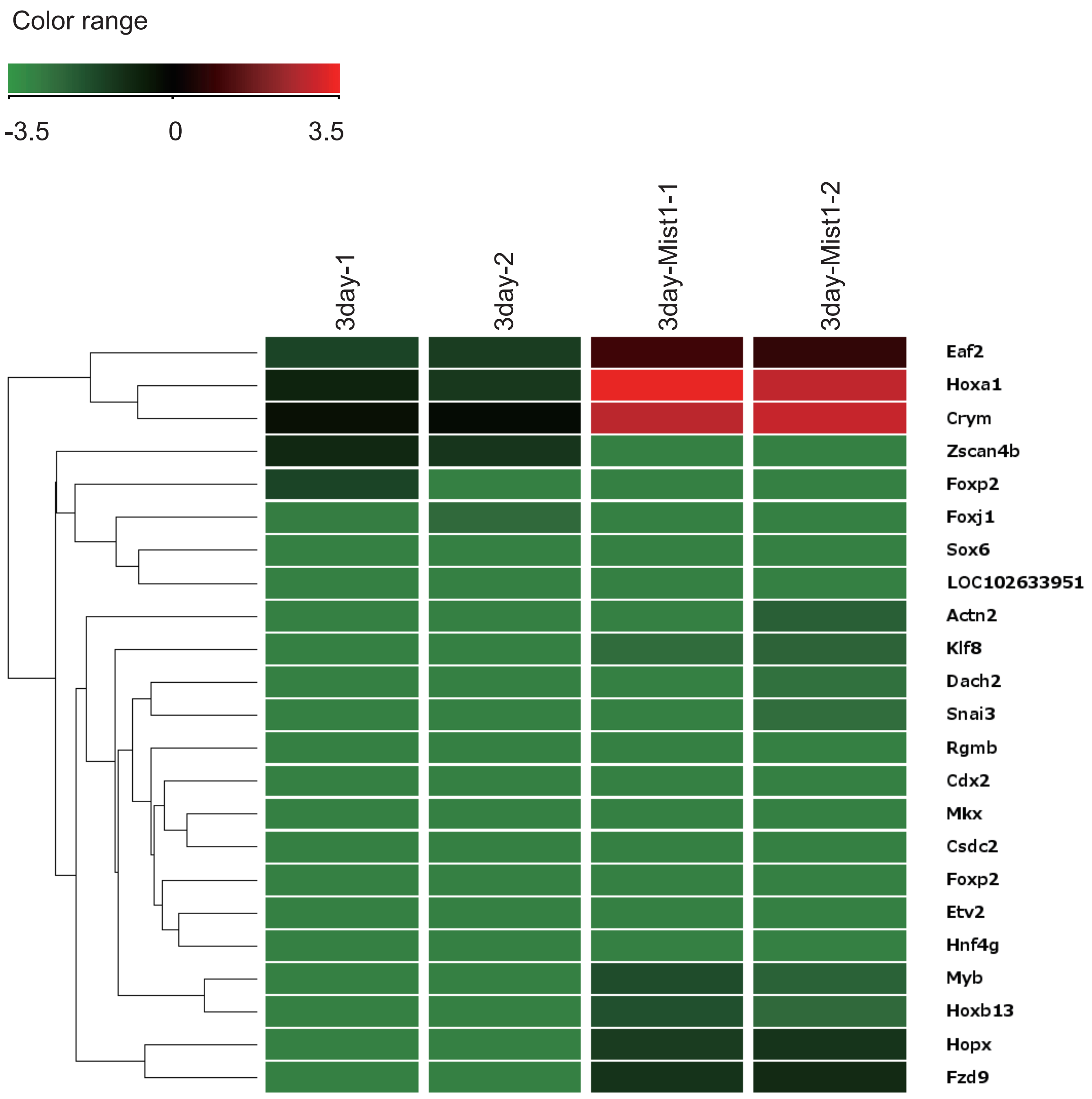

Chikada et al. Supplementary Figure S7

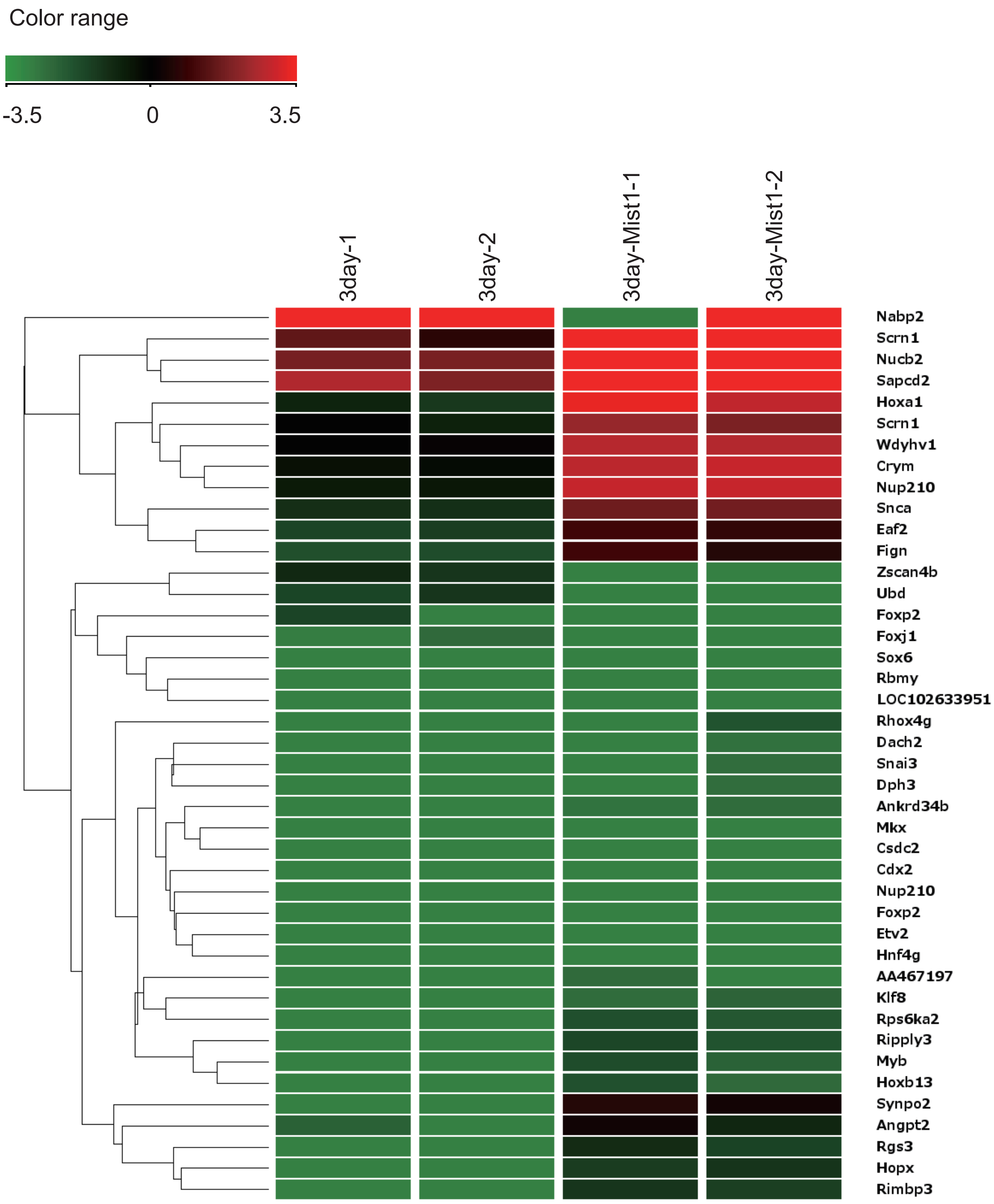

Chikada et al. Supplementary Figure S8

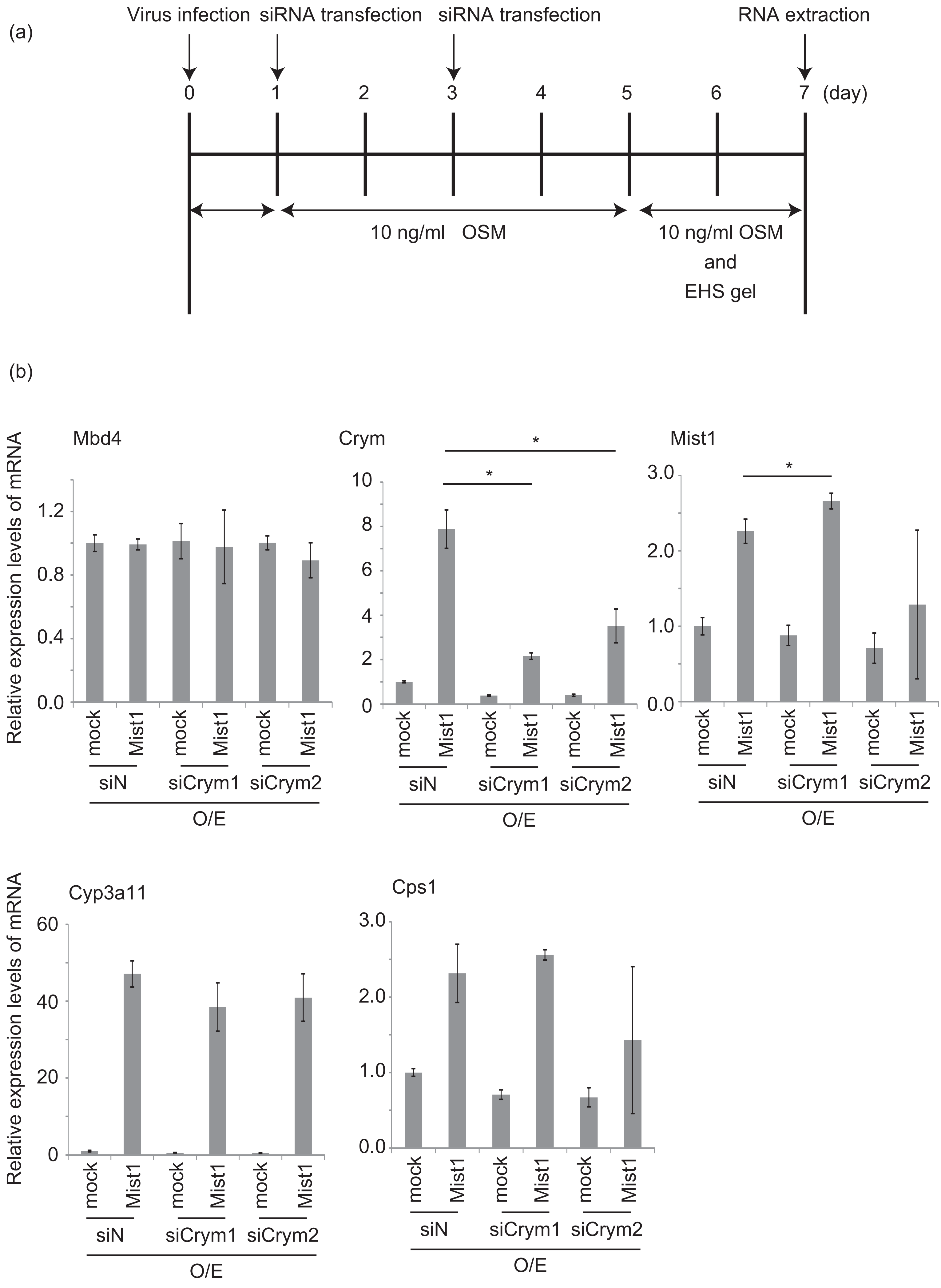

Chikada et al. Supplementary Figure S9

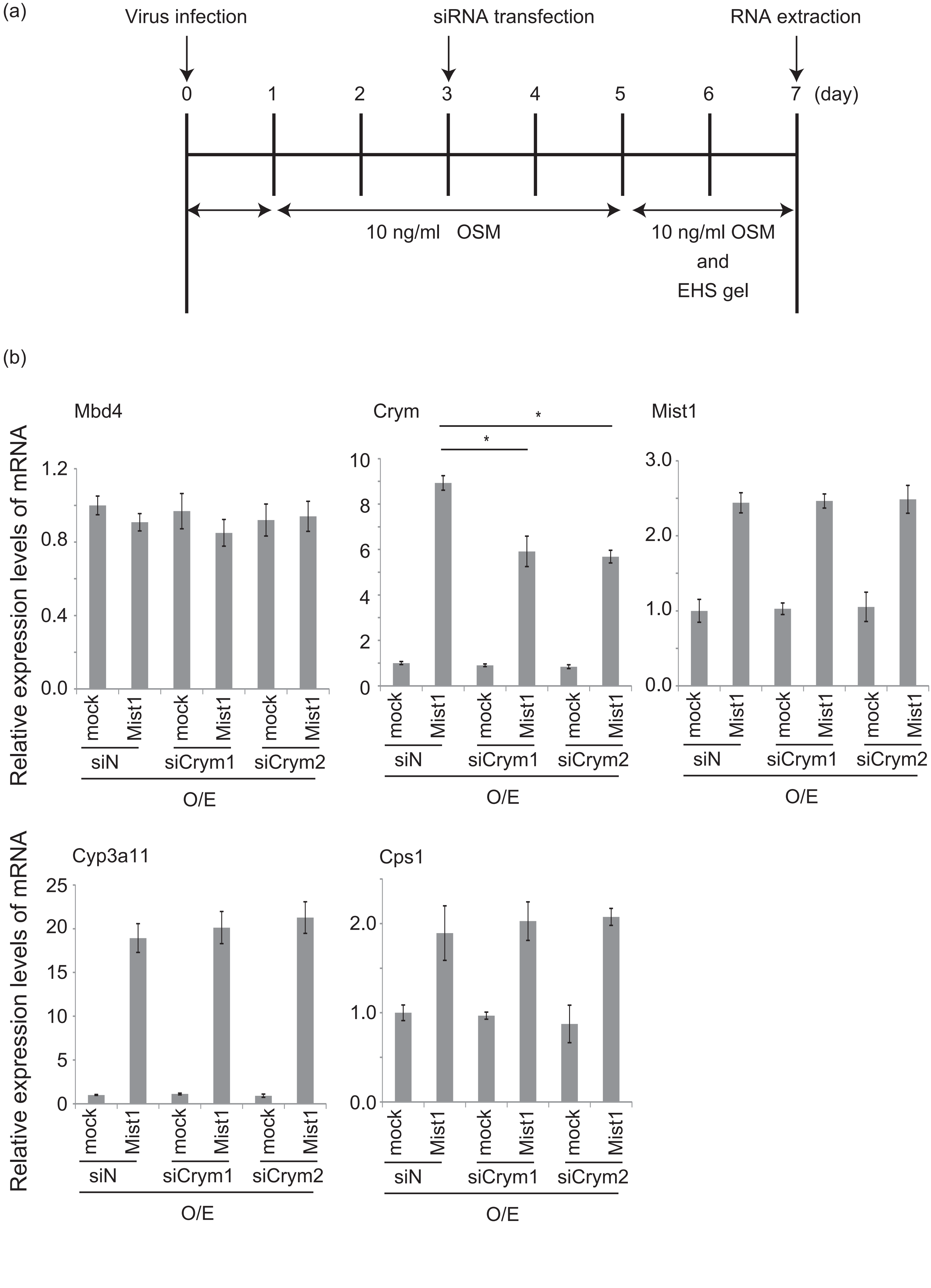

Chikada et al. Supplementary Figure S10
